# Inflammation of the Human Dental Pulp Induces Phosphorylation of eNOS at Thr495 in Blood Vessels

**DOI:** 10.3390/biomedicines10071586

**Published:** 2022-07-03

**Authors:** Özlem Erdek, Wilhelm Bloch, Svenja Rink-Notzon, Hubert C. Roggendorf, Senem Uzun, Britta Meul, Manuel Koch, Jörg Neugebauer, James Deschner, Yüksel Korkmaz

**Affiliations:** 1 Department of Oral and Maxillofacial Plastic Surgery, University of Cologne, 50931 Cologne, Germany; oezlem.erdek@live.de (Ö.E.); hubert.roggendorf@uk-koeln.de (H.C.R.); joerg.neugebauer@zahnarzt-rhein-neckar.de (J.N.); 2 Department of Periodontology and Operative Dentistry, University Medical Center of the Johannes Gutenberg University, 55131 Mainz, Germany; senem.uzun@uni-duesseldorf.de (S.U.); james.deschner@uni-mainz.de (J.D.); 3 Department of Molecular and Cellular Sport Medicine, German Sport University, 50933 Cologne, Germany; w.bloch@dshs-koeln.de (W.B.); bmeul@welldent.de (B.M.); 4 Department of Prosthetic Dentistry, School of Dental and Oral Medicine, University of Cologne, 50931 Cologne, Germany; svenja.rink-notzon@uk-koeln.de; 5 Institute for Experimental Dental Research and Oral Musculoskeletal Biology, Center for Biochemistry, Medical Faculty, University of Cologne, 50931 Cologne, Germany; manuel.koch@uni-koeln.de

**Keywords:** caries, inflammation, dental pulp, oxidative stress, superoxide, peroxynitrite, phosphorylation of eNOS, Ser1177, Thr495, uncoupled eNOS

## Abstract

The activity of endothelial nitric oxide synthase (eNOS) in endothelial cells increased with the phosphorylation of the enzyme at Ser1177 and decreased at Thr495. The regulation of the phosphorylation sites of eNOS at Ser1177 and Thr495 in blood vessels of the healthy and inflamed human dental pulp is unknown. To investigate this, healthy and carious human third molars were immersion-fixed and decalcified. The localization of eNOS, Ser1177, and Thr495 in healthy and inflamed blood vessels was examined in consecutive cryo-sections using quantitative immunohistochemical methods. We found that the staining intensity of Ser1177 in healthy blood vessels decreased in inflamed blood vessels, whereas the weak staining intensity of Thr495 in healthy blood vessels strongly increased in inflamed blood vessels. In blood vessels of the healthy pulp, eNOS is active with phosphorylation of the enzyme at Ser1177. The phosphorylation of eNOS at Thr495 in inflamed blood vessels leads to a decrease in eNOS activity, contributing to eNOS uncoupling and giving evidence for a decrease in NO and an increase in O_2_^−^ production. Since the formation of the tertiary dentin matrix depends on intact pulp circulation, eNOS uncoupling and phosphorylation of eNOS at Thr495 in the inflamed pulp blood vessels should be considered during caries therapy.

## 1. Introduction

A carious lesion is a bacterial infection that leads to inflammation of the dental pulp whenever the caries exceeds the enamel–dentin or cementum–dentin junction and reaches the dentinal tubules. The bacterium *Streptococcus (S) mutans* is considered the main causative agent of caries, whereas, in addition to *S. mutans*, *S. sobrinus, S. downei, Lactobacillus (L) acidophilus, L. casei, L. fermentum, L. rhamnosus, Actinomyces (A) naeslundii* and *A. odontolyticus* are also considered to trigger caries [1,2,3,4,5]. The bacteria reach the dental pulp through the dentinal tubules and induce inflammation by strong vasodilatation and vascular permeability, leading to increased plasma extravasation [6,7,8,9]. In the earlier phase of the carious lesion, the odontoblasts—and after the progression of the lesion, the fibroblasts—endothelial cells, stem cells, and immune cells of the dental pulp recognize bacterial components via pattern-recognition receptors (PRRs) [10,11]. The PRRs, Toll-like receptors (TLRs), and nucleotide-binding oligomerization domain proteins (NOD) 1 and 2 are expressed on odontoblasts, fibroblasts, pulp stem cells, and endothelial cells [10,11]. The binding of bacterial ligands to PRRs leads to activation of transcription factor nuclear factor kappa B (NF-κB) in cells of the dental pulp. Subsequent nuclear translocation of NF-κB as a result of these signaling cascades leads to increased production of cytokines and chemokines (interleukin (IL)-1α and IL-1β; tumor necrosis factor alpha (TNF-α); IL-4, IL-6, IL-8, and IL-10), which trigger cellular immune responses in the dental pulp [10,11]. At the site of inflammation, located near the deepest part of a carious lesion [12], the number of neutrophil granulocytes, macrophages, dendritic cells (DCs), T lymphocytes, B lymphocytes, and plasma cells increases markedly [7,13,14,15]. The recruitment of immune cells and the antibacterial activity of immune cells in the inflamed dental pulp leads to the formation of proteases (matrix metalloproteinases) [16,17], reactive nitrogen species (RNS) [7], and reactive oxygen species (ROS) [18] in the dental pulp. The relatively low physiological concentrations of RNS and ROS are necessary for the regulation of physiological functions of odontoblasts and pulp stem cell differentiation during biomineralization [19]. In inflammation, at higher concentrations, ROS and RNS (superoxide anions, peroxynitrite, hydrogen peroxide, and hydroxyl radicals) cause cellular damage due to their deleterious effects on DNA, proteins, and lipids in cells of the dentin–pulp complex [10,11].

Dependent on the degree of inflammation, local growth factors, neuropeptides, cytokines, and chemokines released by the dissolved dentin matrix, resident pulp cells, and immune cells modulate defense and repair processes to form reactionary or reparative tertiary dentin [10,20]. The reactionary tertiary dentin is formed by the activity of primary odontoblasts in response to inflammation following dentin or deep dentin caries. The reparative tertiary dentin is formed by pulpal stem cells following the death of primary odontoblasts with or without opening of the dental pulp after extremely deep dentin caries [10,11,20,21]. In the treatment of teeth with deep caries and exposed pulp, vital pulp treatment strategies are important to preserve the vitality of the dental pulp [21,22]. When treating a carious lesion with clinical symptoms suggestive of irreversible pulpitis, pulp capping is generally not the treatment of choice [23]. If the dental pulp is exposed to the carious lesion with bacterial inflammation of irreversible pulpitis symptoms [12,24], removal of the inflamed pulp tissue in the form of either a partial pulpotomy [25] or a complete pulpotomy [26,27] is recommended because the pulp in the roots may not be affected by the preoperative inflammation [27]. Since the vitality and formation of reactionary or reparative tertiary dentin depend on an intact circulation of the dental pulp, a thorough understanding of blood vessel regulation in healthy and inflamed dental pulp is essential to develop new treatment approaches in caries therapy and to optimize minimally invasive strategies for the treatment of the dental pulp.

In the endothelium, nitric oxide (NO) is synthesized by the activity of endothelial nitric oxide synthase (eNOS) in a homodimer form of enzymes. In the monomeric form, eNOS is composed of an N-terminal oxygenase and a C-terminal reductase domain. In its N-terminal oxygenase domain, eNOS contains the binding sites for tetrahydrobiopterin (BH4), Zn^2+^, heme, and the substrate L-arginine. In the C-terminal reductase domain, eNOS contains the binding sites for NADPH, FAD, and FMN [28,29,30,31]. Both domains are bound by a CaM domain. The dimeric form of eNOS is linked by Zn^2+^ and coordinated by BH4 of each monomer [30,32]. The interaction with co-factors and binding of the substrate L-arginine only occurs in the dimeric state of eNOS. In the homodimer state, electrons are transferred from NADPH via FAD and FMN in the C-terminal reductase domain of a monomer to the heme in the N-terminal oxygenase domain of the other monomer, where the substrate L-arginine is oxidized by O_2_ to NO and L-citrulline [29,30,33,34].

The activity of eNOS is predominantly regulated by the phosphorylation and dephosphorylation of the enzyme in health and in inflammation [29,30,35,36]. The phosphorylation of eNOS at Ser1177 increases the electron flux within the eNOS homodimer form, resulting in an increase in eNOS activity, while the phosphorylation of eNOS at Thr495 decreases the electron flux within the eNOS homodimer form, inducing a decrease in eNOS activity [29,30]. In inflamed smooth muscle and endothelial cells, NADPH oxidase is stimulated to form O_2_^−^. In inflammation, a higher concentration of NO generated by iNOS and eNOS reacts with O_2_^−^ to form strong oxidant peroxynitrite (ONOO-) [31,33,37,38]. ONOO-oxidizes eNOS essential cofactor BH4 to BH2. BH4 deficiency in eNOS results in uncoupled eNOS, and the activity of uncoupled eNOS leads to O_2_^−^ production instead of NO [31,32,33,37,38,39].

NO produced by eNOS induces vasodilatation of blood vessels, inhibits platelet aggregation and adhesion, prevents leukocyte adhesion to the vascular endothelium and leukocyte migration into the vessel wall, and inhibits vascular smooth muscle cell proliferation [30,37,40,41]. These data suggest several important functions of NO produced by the activity of eNOS in blood vessels under physiological and inflammatory conditions. In the regulation of vascular function in the dental pulp, the bioavailability of NO generated by eNOS activity is essential [42,43]. In blood vessels of healthy rat dental pulp, the phosphorylation site of eNOS at Thr495 was identified [44]. However, the phosphorylation sites of eNOS at Ser1177 and Thr495 in blood vessels of healthy human dental pulp and the effects of dentin carious lesions on the localization of eNOS and on the phosphorylation sites of eNOS at Ser1177 and Thr495 in blood vessels of inflamed human dental pulp are unknown. In the present study, we examined eNOS and phosphorylation sites of eNOS at Ser1177 and Thr495 in blood vessels of healthy and inflamed human dental pulp using quantitative immunohistochemical incubations with specific antibodies.

## 2. Materials and Methods

### 2.1. Tissue Sample Collection, Ethic Statement, and Tissue Preparation

In the present study, due to orthodontics therapy, extracted healthy human (17- to 26-year-old patients’) third molars (*n* = 15) and third molars with dentin caries (*n* = 12) were collected. The molars of the healthy group were unrestored, clinically asymptomatic, and had no pain on percussion. In the inflamed group, the molars showed carious lesions and stimulus-inducible and spontaneous pain with radiographic dentin alterations. The Human Ethics Committee of the Heinrich-Heine University Düsseldorf approved the collection of human third molars.

Immediately after extraction, the molars were immersion-fixed for 24 h in a fixative containing 4% paraformaldehyde and 0.2% picric acid, demineralized in 4 M formic acid, cryoprotected with 30% sucrose solution in 0.1 M PBS pH 7.4, and frozen-sectioned on a cryostat at 30 µm in consecutive sections.

### 2.2. Hematoxylin and Eosin Staining

The healthy and inflamed human third molars were histopathologically characterized by hematoxylin and eosin (H&E) staining, as previously described [45]. The sections were first dried at room temperature for two hours. Subsequently, the sections were washed in distilled water for 5 min and treated with a hematoxylin solution (Carl-Roth GmbH, Karlsruhe, Germany) for 10 min. The sections were washed with slightly warmer running water for 10 min. Following additional washing of the sections with distilled water for 2 min, the sections were treated with eosin solution (Carl-Roth GmbH) for 90 s. The sections were dehydrated in an ascending ethanol series (90%, 95%, 2 × 100% ethanol) for 3 min each. Subsequently, the sections were treated with xylene-I and xylene-II (Carl-Roth GmbH) for 4 min each and coverslipped with Entellan (Merck Millipore, Darmstadt, Germany).

### 2.3. Immunohistochemical Methods

Based on histopathological diagnosis of all collected molars, eight healthy and eight chronically inflamed molars from different patients (*n* = 16) were selected for immunohistochemical incubations. The consecutive sections from each healthy and carious molar were incubated with the antibodies against t-eNOS, Ser1177, and Thr495. Fourth consecutive sections served as controls.

#### 2.3.1. Specificity of the Antibodies

Specificity of antibodies is best characterized by the Western blot method [46,47]. The purified eNOS antibody (sc-654, Santa Cruz Biotechnology) was developed in rabbit against the C-terminus of the human eNOS sequence. The specificity of the eNOS antibody was tested by immunoblot [48]. The rabbit eNOS phosphorylated at Ser1177 antibody directed against a short human amino acid sequence containing Ser1177 (sc-12972-R, Santa Cruz Biotechnology) was characterized by immunoblot [49]. The rabbit eNOS antibody phosphorylated at Thr495 (sc-19827-R, Santa Cruz Biotechnology) was developed against a short human amino acid sequence containing Thr495 and tested by immunoblot [50].

#### 2.3.2. Avidin–Biotin–Peroxidase Complex Method

The free-floating sections were incubated with 0.3% H_2_O_2_ to inhibit endogenous peroxidase. Nonspecific immunoglobulin binding sites were blocked by incubation in 10% normal goat serum (Vector, Burlingame, CA, USA) + 2% bovine serum albumin (Sigma, St. Louis, MO, USA). The sections were incubated overnight with rabbit polyclonal antisera against human eNOS (1:1000; Santa Cruz Biotechnology, Santa Cruz, CA, USA), phospho-eNOS at Ser1177 (1:800; Santa Cruz Biotechnol.), and phospho-eNOS at Thr495 (1:3000; Santa Cruz Biotechnol.) at 4 °C. Then, the sections were incubated for 1 h with biotinylated anti-rabbit IgG (1:500 and 1:1000) (Vector) and for 1 h with avidin–biotin–peroxidase complex (1:100) (Vector). The sections were treated in all incubations for 10 min (to quantify the staining intensities of eNOS, Ser1177, and Thr495 in blood vessels and to statistically analyze the collected data) with a 0.05% 3,3′-diaminobenzidine tetrahydrochloride solution (Sigma) containing 0.05 M Tris-HCl buffer, pH 7.6, 0.01% H_2_O_2_, and 0.01% nickel sulfate. In controls, sections were separately incubated in the absence of the primary antibodies.

#### 2.3.3. Double Immunofluorescence and Confocal Microscopy

The free-floating sections were incubated with guinea pig antibody against human CD31 (Dr. M. Koch, Univ. of Cologne) in 1:1000 dilution overnight at 4 °C. The sections were incubated with DyLight-488-conjugated anti-mouse IgG (or anti-rabbit IgG) (1:1000; Fisher Scientific GmbH, Schwerte, Germany) for 1 h at RT. The sections were then incubated with rabbit antibodies against eNOS (1:1000), phospho-eNOS at Ser1177 (1:800) and phospho-eNOS at Thr495 (1:3000) overnight at 4 °C. Then, the sections were incubated with DyLight-550-conjugated anti-rabbit IgG (1:1000; Fisher Scientific GmbH) for 1 h and with DRAQ5 (1:2000; Cell Signaling Technol.) for 15 min.

Three color fluorescent images were acquired on an LSM 510 META confocal microscope (Carl Zeiss, Jena, Germany). Images were acquired using a 40× oil immersion objective (Carl Zeiss, Jena, Germany) with 633 nm HeNe laser for pseudo-color DRAQ5 nuclear staining, with 488 nm Argon/2 laser for CD31 and with 543 nm laser for eNOS, Ser1177, and Thr495 stainings.

#### 2.3.4. Quantification of Immunohistochemical Results

Immunohistochemical staining intensities of t-eNOS, phospho-eNOS at Ser1177, and phospho-eNOS at Thr495 obtained via the ABC method were quantified by densitometry, as described previously [7]. Morphometric analysis was performed with a Zeiss Axioscop-2 Plus microscope at ×100 magnification coupled with Image System Analysis, Axiovision Ver. 4.7 (Carl Zeiss). In consecutive sections, the staining intensities for eNOS, Ser1177, and Thr495 were measured in three areas of section-free regions (background gray value) and in three areas of the same blood vessels (blood vessel gray value). Then, the mean of the three measured values was calculated for each background and for each blood vessel. The difference between the mean value of the background and the mean value of the blood vessel gave the mean value for each blood vessel of the healthy and inflamed molars (*n* = 8 molars from 8 different patients). The values for the blood vessels of the healthy and inflamed pulp sections were analyzed statistically.

#### 2.3.5. Statistical Analysis

Statistical analyses of the differences between staining intensities of t-eNOS, Ser1177, and Thr495 in blood vessels were compared using Student’s *t*-test or one-way ANOVA with Bonferroni post hoc test used to compare multiple means (SPSS Version 25.0, Chicago, IL, USA). Significance was considered at a *p* value < 0.05.

## 3. Results

### 3.1. Characterization of Healthy and Inflamed Human Dentin–Pulp Complex

**Figure 1:**
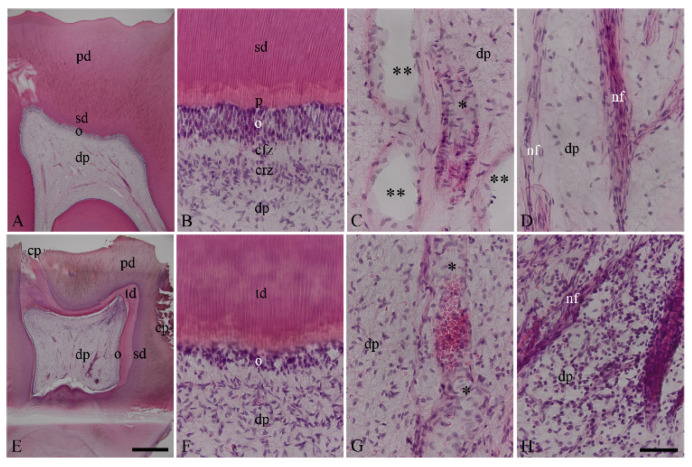
Histopathological characterization of healthy and inflamed human dentin–pulp complex by H&E staining. The cell layers of the healthy dentin–pulp complex are recognizable in a structural order that is characteristic of the histology of the healthy dentin–pulp complex (**A**,**B**). The healthy dentin–pulp complex is recognizable with primary dentin (pd), secondary dentin (sd), predentin (p), the odontoblast layer (o), cell-free zone (cfz), cell-rich zone (crz), and numerous pulp cells (**A**,**B**). The healthy odontoblast layer is visible without degeneration and inflammatory signs (**B**). The venous (two asterisks) and arterial (asterisk) blood vessels and nerve fibers (nf) are seen to not have inflammatory signs (**C**,**D**). The cell layers of the chronically inflamed dentin–pulp complex do not have a structural order (**E**). The chronically inflamed dentin–pulp complex is associated with deep dentin carious lesions in occlusal and in approximal areas (cp). In addition to areas of primary dentin (pd) and secondary dentin (sd), areas of tertiary dentin (td) are found beneath the caries lesions. Several inflammatory cells are found in the dental pulp (**E**). In the odontoblast layer (o), degeneration vacuoles are observed (**F**). In the chronically inflamed dental pulp, dilated blood vessels and nerve fiber are associated with numerous inflammatory cells (**G**,**H**). Scale bar: (**A**,**E**) = 1 mm; (**B**–**D**,**F**–**H**) = 50 µm.

In the healthy dentin–pulp complex (Figure 1A–D), dentin, predentin, the cell layer of odontoblasts, the cell-free and cell-rich regions, subodontoblastic plexus, and dental pulp were identified in a structural order (Figure 1B). The blood vessels were intact, and numerous pulpal stroma cells were distributed around the blood vessels (Figure 1C). The nerve fibers bundles with the accompanying stroma cells branched out regularly in the dental pulp (Figure 1D). In the inflamed dentin–pulp complex (Figure 1E–H), dentin caries triggered an inflammatory response with numerous inflammatory cells in the dental pulp (Figure 1E,H). Beneath the dentin caries, reactive tertiary dentin areas were detected (Figure 1E,F). Blood vessels were dilated, and they contained numerous inflammatory cells and erythrocytes (Figure 1G). Numerous inflammatory cells accumulated in the inflammation region and were distributed around the blood vessels as well as nerve fiber bundles (Figure 1H).

### 3.2. Co-Localization of CD31 with t-eNOS, Ser1177, and Thr495 in Healthy and Inflamed Endothelium of the Human Dental Pulp

**Figure 2:**
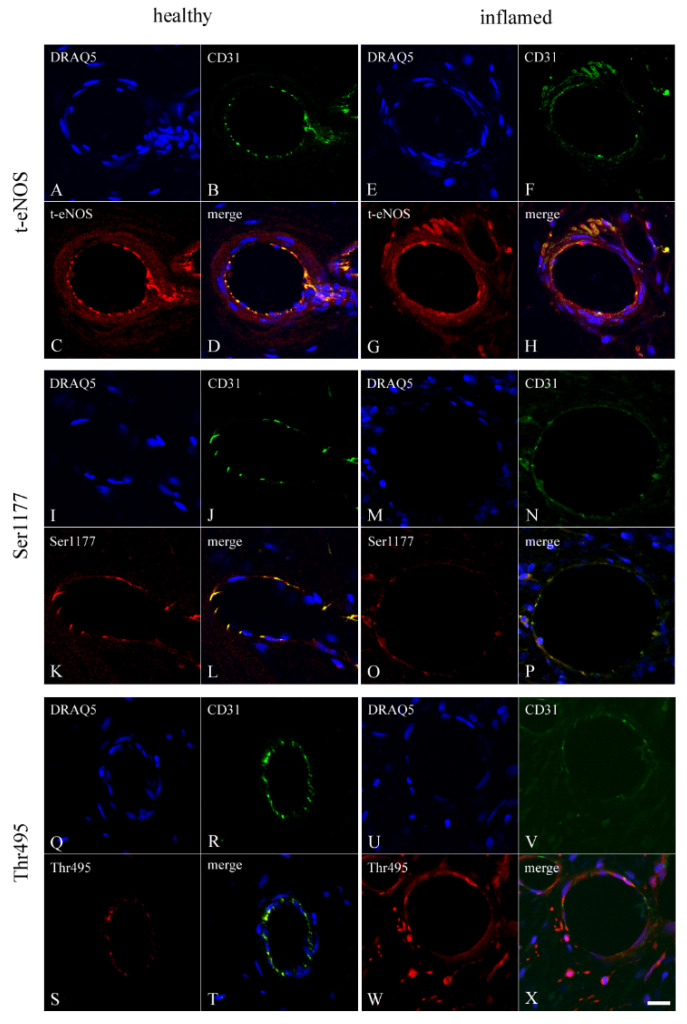
The co-localization of CD31 with eNOS, Ser1177, and Thr495 in blood vessels of the healthy and inflamed human dental pulp. The nuclei of the dental pulp cells are visible by DRAQ5 (a chromosome marker) (**A**). In the endothelium of the pulpal blood vessels, CD31 (a marker for endothelium) (**B**) and t-eNOS (**C**) are co-localized (**D**). The healthy (**C**,**D**) and inflamed (**G**,**H**) vascular smooth muscle cells also show a weak staining intensity for t-eNOS. In capillaries of the inflamed dental pulp, CD31 (**F**) and t-eNOS (**G**) are colocalized (**H**). The cell nuclei of the inflamed dental pulp are identified by DRAQ5 staining (**E**). In endothelium of the inflamed pulpal blood vessels, CD31 (**F**) with a weak staining intensity and t-eNOS (**G**) with a moderate staining intensity are co-localized (**H**). In nuclei of the healthy pulpal cells, DRAQ5 is localized (**I**). CD31 (**J**) and Ser1177 (**K**) are co-localized (**L**) in the endothelium of the healthy dental pulp. The nuclei of cells of the inflamed dental pulp are stained by DRAQ5 (**M**). In endothelium of the inflamed pulpal blood vessels, CD31 (**N**) and Ser1177 (**O**) are co-localized (**P**) with a weak staining intensity. DRAQ5 is identified in nuclei of the healthy pulpal cells (**Q**). In endothelium of the healthy pulpal blood vessels, moderate staining for CD31 (**R**) and weak staining intensity for Thr495 (**S**) was co-localized (**T**). DRAQ5 is detected in nuclei of the inflamed dental pulp cells (**U**). In endothelium of the inflamed pulpal blood vessels, a weak staining for CD31 (**V**) and a strong staining intensity for Thr495 (**W**) are co-localized (**X**). In a subpopulation of the inflamed dental pulp cells, Thr495 is also visible (**W**,**X**). Scale bar: (**A**–**X**) = 20 µm.

To test the localizations of eNOS, Ser1177, and Thr495 in the endothelium, rabbit antibodies against t-eNOS, phospho-eNOS at Ser1177, and at Thr495 were incubated with the guinea pig antibody CD31 (an endothelial marker) using double staining. In healthy (Figure 2A–D) and inflamed (Figure 2E–H) blood vessels of the dental pulp, CD31 and t-eNOS were co-localized in endothelial cells (Figure 2D,H). In the endothelium of healthy (Figure 2I–L) and inflamed (Figure 2M–P) pulp blood vessels, CD31 was co-localized with Ser1177 (Figure 2L,P). The endothelial cells of the healthy (Figure 2Q–T) and inflamed (Figure 2U–X) dental pulp also revealed a co-localization for CD31 with Thr495 (Figure 2T,X).

### 3.3. Localization of t-eNOS, Ser1177, and Thr495 in Healthy Endothelium of the Human Dental Pulp

**Figure 3:**
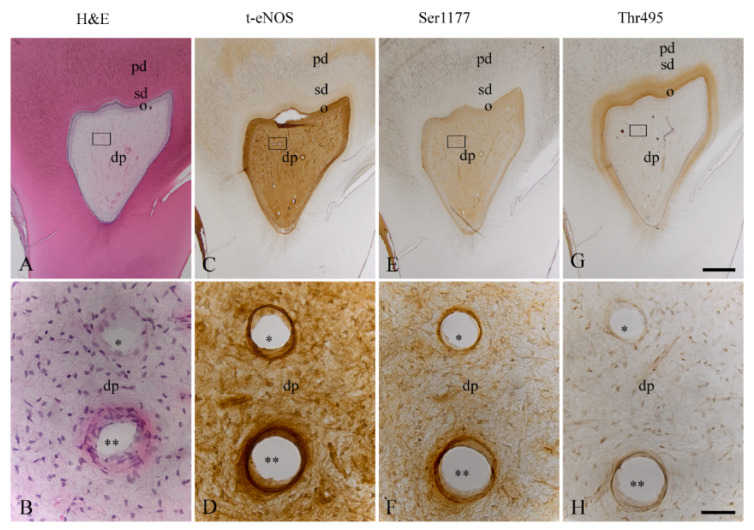
H&E staining and localization of t-eNOS, Ser1177, and Thr495 in blood vessels of the healthy dental pulp of a human third molar. In the overview image of a human third molar, the healthy dentin–pulp complex is found in a structural order with primary dentin, secondary dentin, the odontoblast layer, and the dental pulp without signs of inflammation (**A**). In the detailed image of the region drawn with a rectangle in A, the venous (one asterisk) and arterial (two asterisks) blood vessels and cells in the dental pulp are visible without signs of inflammation (**B**). In the same venous (asterisk) and arterial (two asterisks) blood vessels of the consecutive sections of the healthy dental pulp, eNOS (**C**,**D**) and Ser1177 (**E**,**F**) with stronger staining intensities and Thr495 (**G**,**H**) with weak staining intensity are detected. Odontoblasts show a homogenous localization for eNOS (**C**). Ser1177 (**E**) and Thr495 (**G**) are visible in both the same and different subpopulations of odontoblasts. In some subpopulations of human dental pulp cells, eNOS (**C**,**D**), Ser1177 (**E**,**F**), and Thr495 (**G**,**H**) are also identifiable at different staining intensities. Scale bars: (**A**,**C**,**E**,**G**) = 1 mm; (**B**,**D**,**F**,**H**) = 50 µm.

In the consecutive sections of the healthy dentin–pulp complex (Figure 3A,B), t-eNOS was detected with a strong staining intensity in arterial and venous blood vessels of the dental pulp (Figure 3C,D). In arterial and venous blood vessels of the healthy dental pulp, a strong staining intensity for phospho-eNOS at Ser1177 was detected (Figure 3E,F). In arterial and venous blood vessels of the healthy dental pulp, phospho-eNOS at Thr495 was identified with a weak staining intensity (Figure 3G,H). In comparison to the homogeneous localization of eNOS in odontoblasts (Figure 3C), Ser1177 (Figure 3E) and Thr495 (Figure 3G) were identified with variable staining intensities in both the same and different subpopulations of odontoblasts. In some subpopulations of the dental pulp cells, eNOS (Figure 3C,D), Ser1177 (Figure 3E,F), and Thr495 (Figure 3G,H) were found with different staining intensities.

### 3.4. Localization of t-eNOS, Ser1177, and Thr495 in Inflamed Endothelium of the Human Dental Pulp

**Figure 4:**
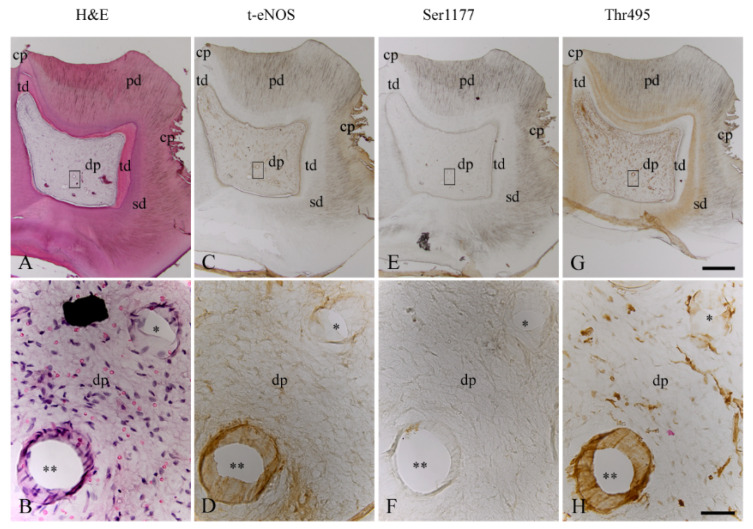
H&E staining and localization of t-eNOS, Ser1177, and Thr495 in blood vessels of the inflamed dental pulp of a human third molar. In the inflamed dentin–pulp complex, occlusal and approximal carious lesions (cp), primary dentin (pd), secondary dentin (sd), and tertiary dentin (td) areas are visible (**A**). In the detailed image of the region drawn with a rectangle in A, the venous (one asterisk) and arterial (two asterisks) blood vessels and inflammatory cells in the dental pulp (dp) are seen (**B**). In the same venous (asterisk) and arterial (two asterisks) blood vessels of the consecutive sections of the inflamed human dental pulp, eNOS (**C**,**D**) with moderate staining intensity, Ser1177 (**E**,**F**) with very weak staining intensity, and Thr495 (**G**,**H**) with strong staining intensity are detected. Odontoblasts of the inflamed dental pulp reveal localizations for eNOS (**C**) and Thr495 (**G**) but not for Ser1177 (**H**). In some subpopulations of the inflammatory dental pulp cells, eNOS (**C**,**D**) and Thr495 (**G**,**H**) are visible at different staining intensities. Scale bars: (**A**,**C**,**E**,**G**) = 1 mm; (**B**,**D**,**F**,**H**) = 50 µm.

In consecutive sections of the inflamed dentin–pulp complex (Figure 4A,B), t-eNOS was detected with a moderate staining intensity in blood vessels (Figure 4C,D). In comparison to the healthy dental pulp (Figure 3E,F), inflammation induced a significant decrease in the staining intensity of phospho-eNOS at Ser1177 in blood vessels (Figure 4E,F). However, compared to the blood vessels of the healthy dental pulp (Figure 3G,H), dentin caries induced a significant increase in the staining intensity of phospho-eNOS at Thr495 in inflamed pulpal blood vessels (Figure 4G,H). In inflamed odontoblasts, eNOS (Figure 4C) and Thr495 (Figure 4G) were detected, whereas Ser1177 (Figure 4E) was not identified in odontoblasts of the inflamed dental pulp. In the inflamed human dental pulp, eNOS (Figure 4C,D) and Thr495 (Figure 4G,H) were identified in some subpopulations of inflammatory cells with different staining intensities. The phosphorylation site of eNOS at Ser1177 (Figure 4E,F) was undetectable in both stromal cells and inflammatory cells of the inflamed dental pulp.

### 3.5. Immunohistochemical Controls

In control incubations of the immunofluorescence double-staining method (Figure S1) and the avidin–biotin–peroxidase complex method (Figure S2), no immunohistochemical staining was found in blood vessels or in other cells of the human dental pulp.

### 3.6. Statistical Analysis of t-eNOS, Ser1177, and Thr495 in Blood Vessels of the Healthy and Inflamed Human Dental Pulp

**Figure 5:**
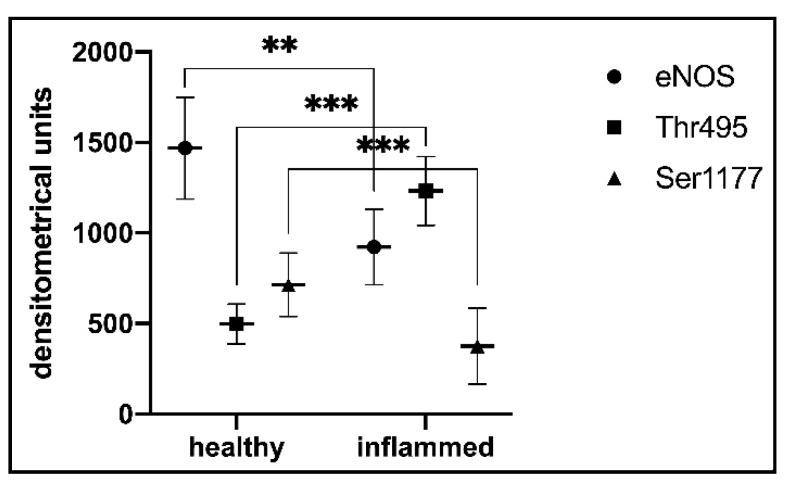
The staining intensities of t-eNOS, Ser1177, and Thr495 in blood vessels of the healthy and inflamed human dental pulp. In both healthy and inflamed pulp blood vessels, eNOS is detected. However, the staining intensity for eNOS in healthy blood vessels is more intense than in inflamed blood vessels. The staining intensity of Ser1177 in healthy blood vessels is significantly higher than in inflamed blood vessels, whereas the weak staining intensity of Thr495 in healthy blood vessels shows a significant increase in inflamed blood vessels. Asterisks indicate significant differences between two compared groups (** *p* < 0.005; *** *p* < 0.0005). For each group, *n* = 8; data are mean ± SD; significant differences were considered at a *p* value <0.05.

In blood vessels of the healthy dental pulp, t-eNOS was detected with a strong staining intensity (*n* = 8; 1468.83 ± 280.94 densitometric units (DU)) (Figure 5). In blood vessels of the inflamed dental pulp, t-eNOS was identified with a moderate staining intensity (*n* = 8; 922.86 ± 207.88 DU) (Figure 5). In blood vessels of the healthy dental pulp, Ser1177 was detected with a strong staining intensity (*n* = 8; 713.59 ± 174.97 DU) (Figure 5). In comparison to the healthy dental pulp, blood vessels of the inflamed dental pulp revealed a weak staining intensity for Ser1177 (*n*= 8; 375.02 ± 210.77 DU) (Figure 5). In blood vessels of the healthy dental pulp, Thr495 was quantified with a weak staining intensity (*n* = 8; 497.32 ± 110.00 DU) (Figure 5). In comparison to the healthy dental pulp, Thr495 was detected with a strong staining intensity in blood vessels of the inflamed dental pulp (*n* = 8; 1232.73 ± 190.49 DU) (Figure 5).

## 4. Discussion

In bacterial inflammation, the knowledge about the expression and the activity of eNOS through its reciprocal phosphorylation sites at Ser1177 and at Thr495 in blood vessels is unknown. The activity of eNOS in endothelial cells increased with the phosphorylation of the enzyme at Ser1177 and decreased at Thr495 [29,30]. Because the blood vessels in the dental pulp are located in a rigid calcified dentin matrix, they have a limited ability to adapt to inflammation in the dental pulp. This unique structure, which does not exist in other organs, clarifies the endothelial function in the dental pulp as even more important. To elucidate the role of eNOS activity in the circulation of the dental pulp during inflammation, we investigated eNOS and phosphorylation sites of eNOS at Ser1177 and Thr495 in blood vessels of healthy and carious human molars using immunohistochemical incubation methods. The comparison of the results from healthy and inflamed pulp blood vessels showed that eNOS in healthy blood vessels phosphorylated strongly at Ser1177 but weakly at Thr495. In the endothelium of inflamed dental pulp, eNOS was weakly phosphorylated at Ser1177 but strongly phosphorylated at Thr495. Our results suggest that inflammation in human dental pulp reduces the activity of eNOS in pulp blood vessels by strongly decreasing the phosphorylation of the enzyme at Ser1177 and strongly increasing the phosphorylation of the enzyme at Thr495.

We found that eNOS is expressed in the blood vessels of both healthy and inflamed pulp, suggesting that eNOS is present at the protein level in the blood vessels of dental pulp under both normal and inflammatory conditions. Superoxide anions (O_2_^−^) are very reactive and a toxic radical and are known to be produced in blood vessels by a reduced form of nicotinamide adenine dinucleotide phosphate (NADPH) oxidases, xanthine oxidase, the mitochondrial respiratory chain, and by uncoupled eNOS [31,41]. NO derived from iNOS or eNOS reacts with O_2_^−^ to form peroxynitrite (ONOO-) [41,51]. Subsequently, BH4 is oxidized by ONOO- to BH2, leading to the uncoupling of eNOS, which then forms O_2_^−^ instead of NO [31,32,39,52,53]. Based on these results, we suggested that inflammation in the dental pulp may induce activity of NADPH-oxidase, leading to increased production of O_2_^−^ and increased oxidative stress in blood vessels of the inflamed dental pulp. In the inflamed human dental pulp, increased activity of iNOS was described [7,42]. The higher concentrations of NO formed by the sustained activity of iNOS and by the activity of eNOS may react with O_2_^−^ in the blood vessels of the inflamed pulp to form ONOO^−^, which has also been detected in higher concentrations in inflamed human dental pulp [7]. It is possible that ONOO^−^ can then oxidize BH4 to BH2, leading to the formation of O_2_^−^ instead of NO due to the higher activity of uncoupled eNOS in the blood vessels of the inflamed dental pulp.

NO-sensitive soluble guanylyl cyclase (sGC), a heterodimeric enzyme with an α- and a β-subunit [54,55], has been characterized in the dentin–pulp complex under physiological and inflammatory conditions [7,43]. In vascular smooth muscle cells (VSMCs), NO binds to the heme of the β_1_-subunit of sGC and activates the enzyme in the reduced Fe^2+^ state under physiological conditions. In heterodimer forms (α_1_β_1_, α_2_β_1_), sGC induces the formation of cGMP in higher concentrations, resulting in vasodilation and suppression of VSMC proliferation [56,57]. In the inflamed dental pulp, ONOO^−^ is formed in higher concentrations [7], so the activity of sGC may be strongly influenced by oxidative stress in VSMCs of the pulp blood vessels. It is known that higher concentrations of O_2_^−^ and ONOO^−^ oxidize sGC (Fe^3+^), resulting in the insensitivity of sGC to NO [58,59]. Additionally, O_2_^−^ and ONOO^−^ enhance the activity of the enzymes phosphodiesterases (PDEs) that degrade cGMP [31,60,61,62]. Inhibition of sGC and degradation of cGMP by PDEs in VSMCs through O_2_^−^ and ONOO^−^ may lead to vascular dysfunction with an increased formation of vasoconstrictors, such as prostaglandin H2 and endothelin 1 [31,63]. In our results, we detected eNOS at the protein levels in blood vessels of the inflamed pulp. Therefore, it is reasonable to assume that vascular dysfunction in human dental pulp caused by higher concentrations of ONOO^−^ and uncoupled eNOS greatly decreases the bioavailability of NO to sGC in blood vessels of the inflamed dental pulp.

NO is involved in the regulation of cell growth, survival, apoptosis, proliferation, and differentiation [56,64]. In healthy odontoblasts, eNOS [43,65], and in inflamed odontoblasts, iNOS and ONOO^−^ [7] have been described. Here, we detected eNOS and Ser1177 in healthy and eNOS and Thr495 in inflamed odontoblasts, supporting our previous findings that the activity of eNOS is increased by the phosphorylation of the enzyme at Ser1177 in healthy treated odontoblasts and decreased at Thr495 in bradykinin-treated odontoblasts [44]. The phosphorylation of eNOS at Thr495 may contribute to the formation of O_2_^−^ in inflamed odontoblasts. At higher concentrations, ONOO^−^ [7] and O_2_^−^ in inflamed odontoblasts result in damage to DNA, proteins, and lipids in cells, leading to their inability to produce extracellular dentin matrix proteins. In some subpopulations of healthy human dental pulp cells, we identified eNOS, Ser1177, and Thr495 with different staining intensities, suggesting that eNOS may be involved in tissue homeostasis by regulating different subpopulations of healthy dental pulp cells through reciprocal phosphorylation at Ser1177 and Thr495. Additionally, we identified the localization of eNOS and Thr495 in a subpopulation of inflammatory cells of the inflamed human dental pulp. It is known that NO can affect T-cell proliferation either positively or negatively, depending on its type, concentration, and source [66]. It has also been found that eNOS can be activated in T helper cells subcellularly in the Golgi apparatus during its translocation to selectively regulate T-cell receptor signaling at the immunological synapse [67]. Based on our results, we speculate that eNOS may be phosphorylated in a subpopulation of immune cells at the Thr495 site to regulate immunological processes. However, to support this hypothesis, further studies are needed to determine the types of these cells using specific markers and to characterize the activity of eNOS at Ser1177 and Thr495 in these cells.

In the present study, we found that eNOS is strongly phosphorylated at Ser1177 in the endothelium of pulpal blood vessels, suggesting the physiological formation of NO by phosphorylation of the enzyme at Ser1177 in the blood vessels of healthy dental pulp. In comparison, the results of the present study showed that eNOS is weakly phosphorylated at Thr495 in the blood vessels of healthy dental pulp. There is evidence showing that eNOS activity can be regulated by dephosphorylation of eNOS at Thr495 with a concomitantantly increased phosphorylation of eNOS at Ser1177 in coordination [68,69] but also without coordination [70]. It is possible that the reciprocal regulation of eNOS activity occurs through strong phosphorylation of the enzyme at Ser1177 and weak phosphorylation of the enzyme at Thr495 in blood vessels of the healthy dental pulp to coordinate the formation of physiological NO concentration in response to various stimuli. In comparison to other tissues, the dental pulp has a higher intra-pulpal tissue pressure due to the calcified rigid dentin matrix [71,72,73,74,75,76]. The endothelial cells are mechanosensitive [77,78], and it is known that phosphorylation of eNOS at Ser1177 is increased by shear stress [79]. Because the dental pulp has higher physiological intrapulpal tissue pressure compared with other tissues [75,76], it is possible that higher physiological intrapulpal tissue pressure causes an increase in shear stress due to increased friction between blood flow and the endothelium, leading to strong phosphorylation of eNOS at Ser1177. However, further studies need to clarify whether higher physiological intrapulpal tissue pressure can regulate the phosphorylation of eNOS at Ser1177.

In blood vessels, NADPH oxidase is activated by protein kinase C (PKC) [31,62]. The increased activity of NADPH oxidase leads to a greater formation of O_2_^−^ and ONOO^−^, which oxidize BH4 to BH2 in eNOS, resulting in the uncoupling of eNOS [31,32,39,62]. Uncoupled eNOS produces O_2_^−^ instead of NO [31,38,41,62]. It was reported that O_2_^−^ and ONOO^−^ also trigger the activation of PKC [31,62]. In blood vessels, the activity of eNOS is reduced by the activity of PKC due to the strong phosphorylation of the enzyme at Thr495 and dephosphorylation of the enzyme at Ser1177, which can lead to a strong reduction in the formation of NO [69]. It has been described that agonists that increase intracellular calcium can dephosphorylate eNOS at Thr495 through protein phosphatases PP1, PP2A, or PP2B [69,80]. Mimicking the dephosphorylation of eNOS at Thr495 by mutating the site to alanine results in the uncoupling of eNOS [81]. These results show that the uncoupling of eNOS by phosphorylation of the enzyme at Thr495 leads to increased production of O_2_^−^ instead of NO [82]. In our results, the activity of eNOS is strongly reduced by the decreased phosphorylation of eNOS at Ser1177 and by the increased phosphorylation of the enzyme at Thr495 in blood vessels of the inflamed dental pulp. Based on these results, we suggested that eNOS in inflamed blood vessels is potentially uncoupled by ONOO^−^ through the oxidation of BH4 to BH2. The activity of eNOS in blood vessels of the inflamed dental pulp may be reduced by the uncoupling eNOS and by strong phosphorylation of the enzyme at Thr495 inducing the formation of O_2_^−^ instead of NO. In a rigid dentin matrix room, inflammation additionally increases intrapulpal tissue pressure, resulting in a strong reduction in pulpal blood flow via the apical foramen to the dental pulp [73,74,75,76]. Therefore, we suggest that the decrease in pulpal blood flow led to a decrease in shear stress in pulp blood vessels, resulting in a decrease in eNOS phosphorylation at Ser1177 but a strong increase in phosphorylation at Thr495. Our suggestion is supported by results that revealed that low shear stress induced the phosphorylation of eNOS at Thr495 by activation of MAPK ERK1/2 [82,83]. It was also described that low shear stress is associated with inflammation of the endothelium and that oxidative stress reduces the activity of eNOS [82,83,84,85]. Based on our results, we hypothesize that the morphological characteristics of an organ may determine its tissue pressure status. These organ-specific tissue pressure states, associated with changes in physiological and inflammatory conditions, may cause changes in blood flow and thus changes in shear stress in blood vessels regulating the phosphorylation of eNOS at Ser1177 and Thr495 in an organ-specific manner. Further studies are needed to clarify whether the inflammation-induced increase in intrapulpal tissue pressure and the decrease in pulpal blood flow regulate the phosphorylation of eNOS at Ser1177 and Thr495 by PKC.

The dental pulp is supplied by end arteries and veins through the apical foramen [86,87,88], located in a rigid calcified extracellular dentin matrix, and has a limited ability to expand in response to inflammation induced by caries [73,74,75,76]. The objective of caries therapy is to induce the formation of the reactionary and reparative tertiary dentin matrix in response to inflammation in the dental pulp [11,89]. Increased ROS formation at the site of inflammation causes endothelial dysfunction and leads to a decrease in antioxidant endogenous defense activity [90]. It has been reported that the reduction in inflammatory responses by antioxidant enzymes in rat dental pulp leads to a significant formation of the tertiary dentin matrix [91]. Since the formation of the tertiary dentin matrix depends on intact pulp circulation, the pulp vascular dysfunction by uncoupled eNOS and by phosphorylation of eNOS at Thr495 in the inflamed pulp due to the formation of O_2_^−^ and ONOO^−^ may impair the formation of a reactionary and reparative tertiary dentin matrix and promote the development and persistence of carious lesions. Thus, in inflammation of the dental pulp, the formation of bioavailable NO in pulp blood vessels could be promoted by the local pharmacological manipulation of eNOS phosphorylation at Ser1177 and Thr495 by pulp-capping agents, which would allow the development of new vital pulp therapy strategies for caries treatment.

## 5. Conclusions

In the present study, we detected eNOS in blood vessels of both healthy and inflamed dental pulp, suggesting that eNOS is present in blood vessels of both healthy and inflamed blood vessels and that the posttranslational activity of eNOS may be regulated by phosphorylation of the enzyme. Our results show that the activity of eNOS in blood vessels of the healthy dental pulp is increased by strong phosphorylation of the enzyme at Ser1177 but weak phosphorylation at Thr495. In comparison, the activity of eNOS in blood vessels of the inflamed dental pulp is reduced by weak phosphorylation of the enzyme at Ser1177 but strong phosphorylation of the enzyme at Thr495. We conclude that phosphorylation of eNOS at Thr495 in inflamed blood vessels leads to a decrease in eNOS activity, which may contribute to eNOS uncoupling, and provides evidence for a decrease in NO and an increase in O_2_^−^ production. The organ-specific tissue pressure changes in health and inflammation alter the signaling behavior of endothelial cells regulating the phosphorylation of eNOS.

## Data Availability

Data sharing is not applicable to this article.

## Supplementary Materials

The following supporting information can be downloaded at: https://www.mdpi.com/article/10.3390/biomedicines10071586/s1, Figure S1: Immunohistochemical controls of the avidin-biotin-peroxidase method in the blood vessels of human healthy dental pulp; Figure S2: Immunohistochemical controls of the immunofluorescence double staining method in the blood vessels of human healthy dental pulp.
