# Supplementary material for: Inflammation of the Human Dental Pulp Induces Phosphorylation of eNOS at Thr495 in Blood Vessels
Source: Biomedicines. 2022 Jul 3;10(7):1586. doi: 10.3390/biomedicines10071586 (PMC9313222; doi:10.3390/biomedicines10071586)
Supplement: Supplementary file 1 [file biomedicines-10-01586-s001.zip › biomedicines-1774288-supplementary-1.pdf]

Supplementary Materials

Article

Inflammation of the human dental pulp induces phosphorylation of eNOS at Thr495 in blood vessels

Özlem Erdek [1,2], Wilhelm Bloch [3], Svenja Rink-Notzon [4], Hubert C. Roggendorf [1], Senem Uzun [2], Britta Meul [3], Manuel Koch [5], Jörg Neugebauer [1], James Deschner [2] and Yüksel Korkmaz [2,*]

1 Interdisciplinary Department of Oral Surgery and Implantology, Department of Craniomaxillofacial and Plastic Surgery, University of Cologne, Cologne, Germany; oezlem.erdek@live.de; hubert.roggendorf@uk-koeln.de; joerg.neugebauer@zahnarzt-rhein-neckar.de
2 Department of Periodontology and Operative Dentistry, University Medical Center of the Johannes Gutenberg University, Mainz, Germany; senem.uzun@uni-duesseldorf.de; james.deschner@uni-mainz.de; yueksel.korkmaz@unimedizin-mainz.de
3 Department of Molecular and Cellular Sport Medicine, German Sport University, Cologne, Germany; w.bloch@dshs-koeln.de; bmeul@welldent.de
4 Department of Prosthetic Dentistry, School of Dental and Oral Medicine, University of Cologne, Cologne, Germany; svenja.rink-notzon@uk-koeln.de
5 Institute for Experimental Dental Research and Oral Musculoskeletal Biology, Center for Biochemistry, Medical Faculty, University of Cologne, Cologne, Germany; manuel.koch@uni-koeln.de
* Correspondence: yueksel.korkmaz@unimedizin-mainz.de; Tel.: +49-6131-17-7247

S1: Immunohistochemical Controls:

To test the specificity of the immunohistochemical reagents (secondary antibodies, avidin-biotin-peroxidase complex, NGS, BSA, DAB), sections were separately incubated in the absence of the primary antisera. In control incubations, no immunohistochemical staining was found in blood vessels or in other cells of human dental pulp (Figure S1, Figure S2).

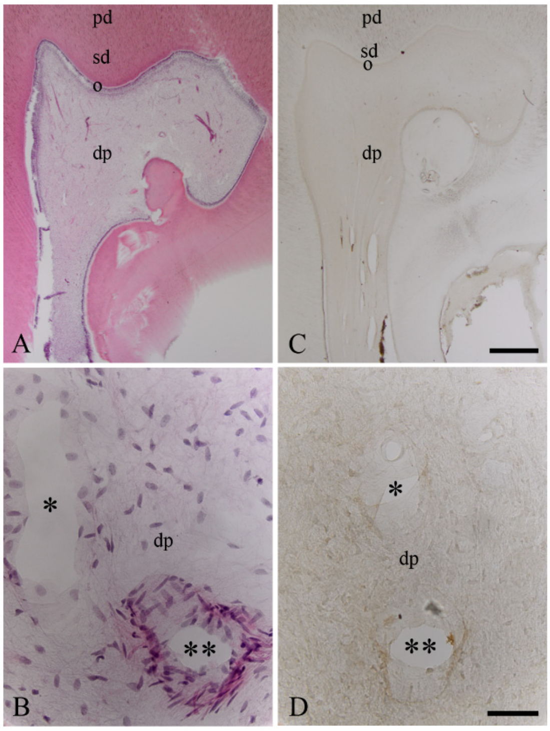

Figure S1. Immunohistochemical controls of the avidin-biotin-peroxidase method in the blood vessels of human healthy dental pulp. In the overview image of the healthy dentin-pulp complex, primary dentin, secondary dentin, the odontoblast layer and the dental pulp are visible in a structural and cellular order (A). Numerous cells of the dental pulp can be seen in the detailed image (B). The veins (one asterisk) and arteries (two asterisks) are identified in the healthy dental pulp (B). In the control incubations, where the first antibodies were omitted, the immunohistochemical staining in the blood vessels of the healthy dental pulp is not visible in the overview (C) and detail (D) images. pd=primary dentin, sd=secondary dentin, o=odontoblasts, dp=dental pulp. Scale bars: A,C= 1 μm, B, D= 50 μm.

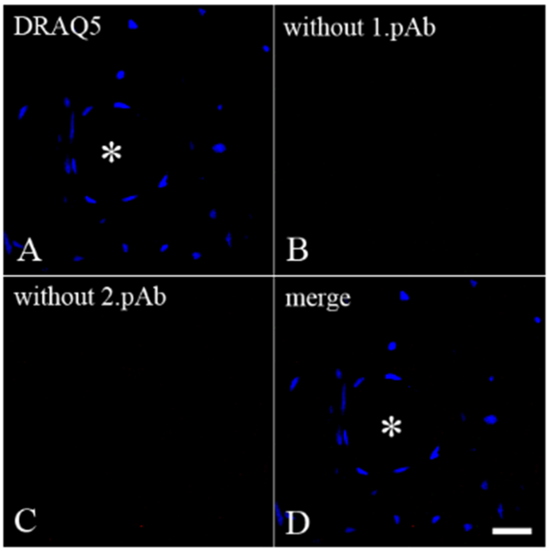

Figure S2. Immunohistochemical controls of the immunofluorescence double staining method in the blood vessels of human healthy dental pulp. DRAQ5 (a chromatin marker) is found in cell nuclei of endothelial cells of blood vessels (asterisk) and cells of healthy dental pulp (A). In the control incubations in which first primary (1.pAb) and second primary (2.pAb) antibodies were omitted from the incubations, an immunohistochemical staining in blood vessels was not evident (C, D). Scale bar: A-D= 20 μm.
